# Genome-Wide Analysis of Heat Shock Transcription Factors (HSFs) in Kelp (*Saccharina japonica*) and Analysis of Their Expression in Response to Abiotic Stresses

**DOI:** 10.3390/plants15030429

**Published:** 2026-01-30

**Authors:** Wentai Mao, Wenbo Zhu, Ruixue Li, Jianjun Lu, Yijuan Han, Weiqi Tang, Hongmei Lin, Wenshan Wang, Xiaoting Chen, Songbiao Chen, Wenwei Lin, Zhongyuan Lin

**Affiliations:** 1 College of Life Sciences, Fujian Agriculture and Forestry University, Fuzhou 350002, China; 2 Fujian Provincial Universities Engineering Research Center of Marine Biology and Drugs, College of Geography and Oceanography, Minjiang University, Fuzhou 350108, China; 3 Fujian Key Laboratory on Conservation and Sustainable Utilization of Marine Biodiversity, College of Geography and Oceanography, Minjiang University, Fuzhou 350108, China; 4 Fuzhou Technological Innovation Center of Seawater Planting Industry, Minjiang University, Fuzhou 350108, China; 5 Fujian Key Laboratory of Agroecological Processing and Safety Monitoring, Fujian Agriculture and Forestry University, Fuzhou 350002, China

**Keywords:** *Saccharina japonica*, brown algae, heat shock transcription factors (HSFs), heat stress, yeast one-hybrid

## Abstract

Heat shock transcription factors (HSFs) play a crucial role in mediating responses to abiotic stresses. However, characterization of HSFs in macroalgae remains largely unexplored. In this study, a comprehensive analysis of HSFs was carried out in *Saccharina japonica*. A total of sixteen *SjHSFs* were identified. Phylogenetic analysis revealed that HSFs from brown algae form a distinct clade, separate from those from red algae, green algae, moss, and *Arabidopsis thaliana*. The DNA-binding domain was found to be highly conserved among SjHSFs. Analysis of cis-acting elements in *SjHSF* promoters suggested their potential roles in regulating growth, development, and stress responses. Tissue-specific expression profiles revealed differential expression of *SjHSFs* across various tissues of *S. japonica*. Under abiotic stresses, certain *SjHSFs* exhibited dynamic expression patterns, with particularly pronounced changes observed under high-temperature stress. We further employed a transcription factor-centered yeast one-hybrid (TF-Centered Y1H) to determine the motifs recognized by SjHSF-03. Seven conserved motifs were identified, and the distributions of these motifs were screened in the promoter regions of *S. japonica* genes involved in diverse biological processes and pathways. Notably, 23 heat shock protein (*HSP*) genes were among these motif-containing genes, and 21 out of these 23 *SjHSPs* were up-regulated under heat stress. Our results provide a solid foundation for future research on the specific functions of HSFs under different stress conditions and the regulatory mechanisms of HSF-mediated stress responses in *S. japonica* and other brown algae.

## 1. Introduction

Algae, as well as other organisms (e.g., plants and animals) are constantly exposed to various environmental stresses, including high temperature, heavy metals, and light stress. Among these, high temperature particularly impacts algae′s reproductive development [1,2,3]. Through natural evolution, these organisms have developed adaptive strategies to cope with environmental stresses, such as the regulation of gene/protein expression to modulate metabolic pathways for heat stress adaptation. In the heat shock signal transduction pathway, heat shock transcription factors (HSFs) are the terminal components and primary regulators of the heat shock (HS) response, controlling the expression of downstream heat shock protein (*HSP*) genes [4].

The *HSF* gene was initially cloned in *Drosophila* and subsequently reported in yeast and mammals [5,6]. The first report of plant HSF was from tomatoes under heat stress [7]. Compared to animals, plant HSF family members showed greater diversity in quantity, structure, and regulatory mechanisms [7]. For example, the *Arabidopsis thaliana* HSF family comprises 21 members, including 15 belonging to class A, five to class B, and one to class C [8]. HSFs have a highly conserved structure, typically containing five major domains, namely the DNA-binding domain (DBD), oligomerization domain (OD), nuclear localization signal (NLS), nuclear export signal (NES), and C-terminal transcriptional activation domain (CTAD) [9]. The oligomerization domain includes HR-A and HR-B regions, which are used to classify three types of HSF: HSFA, HSFB, and HSFC. HSFA is characterized by an AHA motif and functions as a transcriptional activator, whereas class B/C HSFs lack this motif and are transcriptionally inactive.

HSFs are core regulatory factors that play crucial roles in enhancing thermotolerance and other stress resistance mechanisms in plants. Currently, research on HSFs has primarily focused on class A and class B, with relatively few studies on class C. Generally, HSFA members can activate transcription and confer stress resistance abilities to plants (e.g., heat tolerance), while HSFB members act as repressors or coactivators of HSFA members [10,11,12,13,14]. HSFA members serve as direct regulators of heat stress and other stress responses. Interestingly, recent studies have indicated that *HSFB* genes may also directly and positively regulate stress resistance in plants [2,15,16]. Among algae, *HSF* genes have been reported in the green algae *Chlamydomonas reinhardtii*, the red algae *Neopyropia yezoensis*, and the diatom *Phaeodactylum tricornutum* [2,17,18]. *C. reinhardtii* contains a single canonical HSF (HSF1) that exhibits typical features of plant class A HSFs and serves as a key regulator of stress responses [16]. In *N. yezoensis*, *NyHSF* responds to high temperatures and shows tissue-diverse expression patterns [18]. In *P. tricornutum*, PtHSF1 upregulates glycerol-2phosphate acyltransferase 3 (GPAT3) and 1-deoxy-d-xylulose 5-phosphate synthase (DXS) by directly binding to their promoters; genetic analysis further demonstrates that PtHSF1 acts upstream of GPAT3 and DXS to regulate triacylglycerol and fucoxanthin synthesis [19].

*Saccharina japonica*, a member of the class Phaeophyceae, order Laminariales, family Laminariaceae, and genus *Saccharina*, is a species native to cold temperate zones with an optimal growth temperature of 10 to 15 °C. It is currently the large seaweed with the largest cultivation area and highest yield in China [20]. *S. japonica* culture has the potential to contribute significantly to both economic and environmental sustainability [21]. Although *S. japonica* is now farmed in the southern regions of China, high temperature remains an important factor affecting its growth, development, and geographical distribution [22]. Previous studies have reported that each 1 °C increase in temperature significantly reduces the biomass and the reproductive capacity of spores of *S. japonica* [21]. In this study, we conducted a genome-wide identification and characterization of the *HSF* genes in *S. japonica*. Transcriptome datasets were further integrated to dissect the expression profiles of the *SjHSF* genes across various developmental tissues and under adverse stress conditions. Additionally, a transcription factor-centered yeast one-hybrid (TF-Centered Y1H) assay was employed to identify the DNA motifs specifically recognized by SjHSF-03. Screening of these motifs in the promoter regions of *S. japonica* genes revealed their widespread distribution among genes involved in diverse biological processes and signaling pathways.

## 2. Results

### 2.1. Identification of HSF Genes in S. japonica

**Figure 1:**
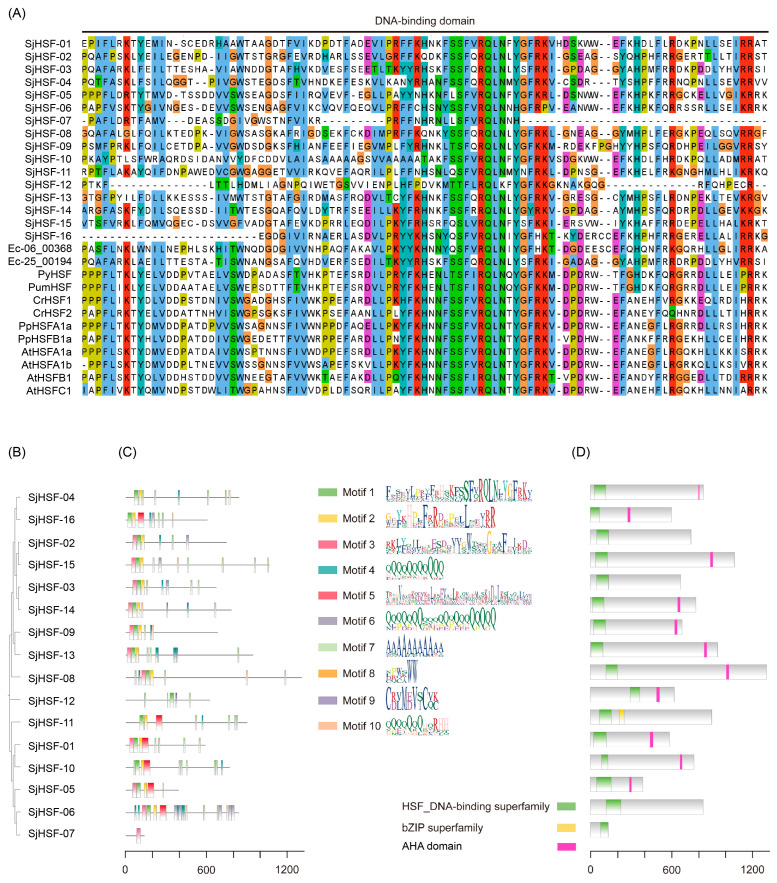
DNA binding domain (DBD), phylogenetic tree, protein motif, and conserved domain analysis. (**A**) Amino acid sequences of the DBD regions of SjHSFs and *E. siliculosus*, *P. yezoensis*, *P. umbilicalis*, *C. reinhardtii*, *P. patens*, and *A. thaliana* HSF family members were aligned. The DBD is indicated with one line. (**B**) The maximum likelihood phylogenetic tree of SjHSFs. (**C**) The conserved motifs in SjHSFs. Different motifs and their relative positions are represented by the colored boxes. (**D**) The HSF DNA-binding domain in SjHSFs. The scale at the bottom can be used to estimate domain length.

To identify the SjHSFs, we used the HSF domain (PF00447) as the query to search the *S. japonica* genome [23,24]. Finally, sixteen HSF-like proteins were identified and designated SjHSF01–SjHSF16. The lengths of putative *SjHSF* CDS ranged from 411 bp to 3978 bp, and their corresponding protein sequences ranged from 136 amino acids (SjHSF-07) to 1325 amino acids (SjHSF-08) (Table 1 and Table S1). The molecular weights varied from 14.75 kDa (SjHSF-07) to 131.25 kDa (SjHSF-08), while the theoretical pIs ranged from 4.98 (SjHSF-01) to 10.82 (SjHSF-12). The instability index values spanned 38.26 (SjHSF-11) to 79.62 (SjHSF-07). Bioinformatic predictions of subcellular localization indicated that all SjHSFs localize to the nucleus (Table 1). Multiple sequence alignment revealed that the DBD regions within SjHSFs were highly conserved (Figure 1A).

To characterize the structural features of SjHSFs, we analyzed their conserved motifs and domains (Figure 1B–D). Ten distinct motifs were identified across all SjHSFs (Figure 1C), and the motif combinations can be grouped as three categories (Figure 1B,C). The first category all contained motif 1, motif 2 and motif 3, which correspond to DBD, including SjHSF-02, -03, -08, -09, -13, -14, and -15. The second category (SjHSF-01, -05, -06, -07, -10, -11, and -16) primarily featured motif 3 or motif 5, especially, SjHSF-07 retained only one motif. The third category comprised only SjHSF-12. All SjHSFs harbored HSF DNA-binding superfamily domain (Figure 1D). Notably, SjHSF-11 encodes an additional basic leucine zipper (bZIP) domain (Figure 1D). Furthermore, eleven SjHSFs contained the AHA motif, a defining feature of HSFA (Figure 1D, Table S2). However, there is no specific HR-A/B region characteristics in SjHSFs (Figure S1). It is only possible to vaguely discern the boundary of HR-A within SjHSFs, while the characteristic features of HR-B are absent (Figure S1).

### 2.2. Gene Structure and Chromosomal Distribution of SjHSFs

**Figure 2:**
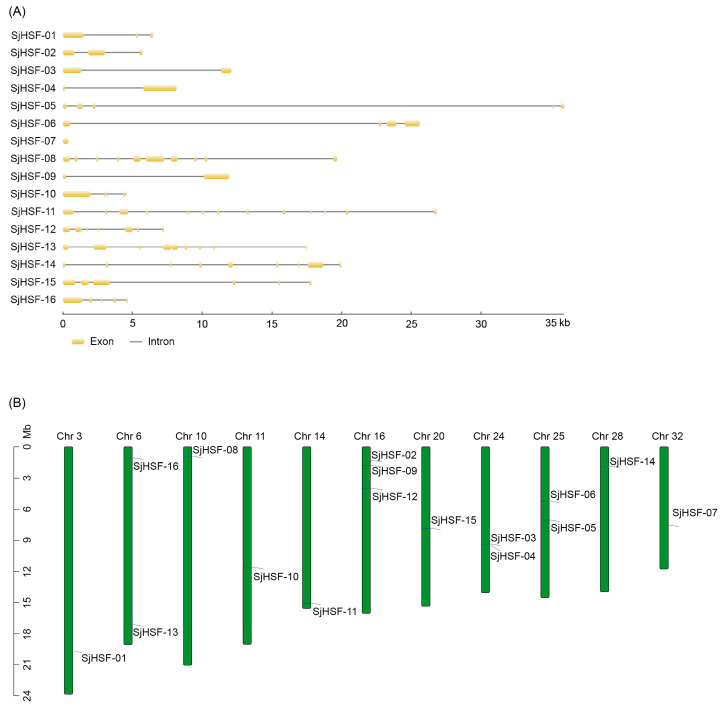
Gene structures and chromosomal location of *HSFs* in *S. japonica*. (**A**) Exon/intron structures of *SjHSFs*. Yellow boxes represent exons and black lines represent introns. The sizes of exons and introns can be estimated using the scale at the bottom. (**B**) Distribution of *SjHSF* genes in the kelp genome. Note: Chr = Chromosome.

The exon/intron structure of *SjHSFs* was determined by aligning the genomic sequence against the corresponding CDS. With the exception of *SjHSF-07*, most *SjHSFs* contained introns. The number of exons per *SjHSF* gene ranged from one to twelve (Figure 2A): *SjHSF-03*, *-04*, and *-09* each contained a single intron; *SjHSF-01* and *-02* each contained two introns; *SjHSF-05*, *-10*, and *-16* each contained four introns; and *SjHSF-11* had a maximum of twelve. The chromosomal locations of *SjHSFs* were confirmed using genome annotation data [23,24]. The 16 *SjHSFs* were mapped to chromosomes, with an uneven distribution (Figure 2B). Chromosomes 3, 10, 11, 14, 20, 28, and 32 each had only one gene, while chromosomes 6, 24, and 25 contained two and three genes, respectively. Three *SjHSFs* were distributed on chromosome 16.

### 2.3. Phylogenetic Analysis of SjHSFs

**Figure 3:**
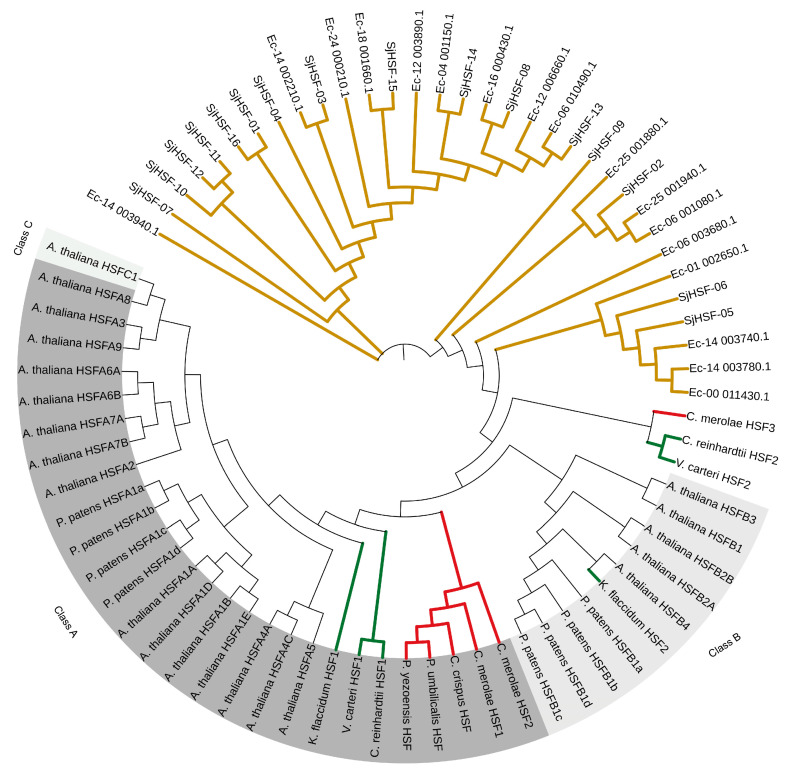
Phylogenetic analysis of HSF proteins from *S. japonica* and multiple species. Phylogenetic tree of HSFs from *E. siliculosus* (17), *P. yezoensis* (1), *P. umbilicalis* (1), *C. merolae* (3), *C. crispus* (1), *C. reinhardtii* (2), *V.carteri* (2), *K. flaccidum* (2), *P. patens* (8), *A. thaliana* (21), and *S. japonica* (16). The phylogenetic tree was constructed using the Maximum Likelihood (ML) method with 1000 bootstrap replications. Red lines: Red algae; Green lines: Green algae; Brown lines: Brown algae.

To investigate the evolution of SjHSFs, a phylogenetic tree was constructed using HSF sequences from representative species, including a brown alga (*E. siliculosus*), four red algae (*P. yezoensis*, *P. umbilicalis*, *C. merolae*, and *C. crispus*), three green algae (*C. reinhardtii*, *V. carteri*, and *K. flaccidum*), *P. patens*, and *A. thaliana* (Figure 3). Phylogenetic analysis revealed that HSFs from brown algae formed a distinct evolutionary cluster, with HSFs from other species clustering separately (Figure 3). Notably, *K. flaccidum*, *P. patens*, and *A. thaliana* were classified into a separate clade encompassing both HSF class A and class B members (Figure 3). HSFs from green algae (*C. reinhardtii* and *V. carteri*) and all four red algae were exclusively classified as class A, whereas *C. reinhardtii* HSF2, *V. carteri* HSF2, and *C. merolae* HSF3 diverged from class A and class B.

### 2.4. Cis-Regulatory Element Analysis of SjHSFs

**Figure 4:**
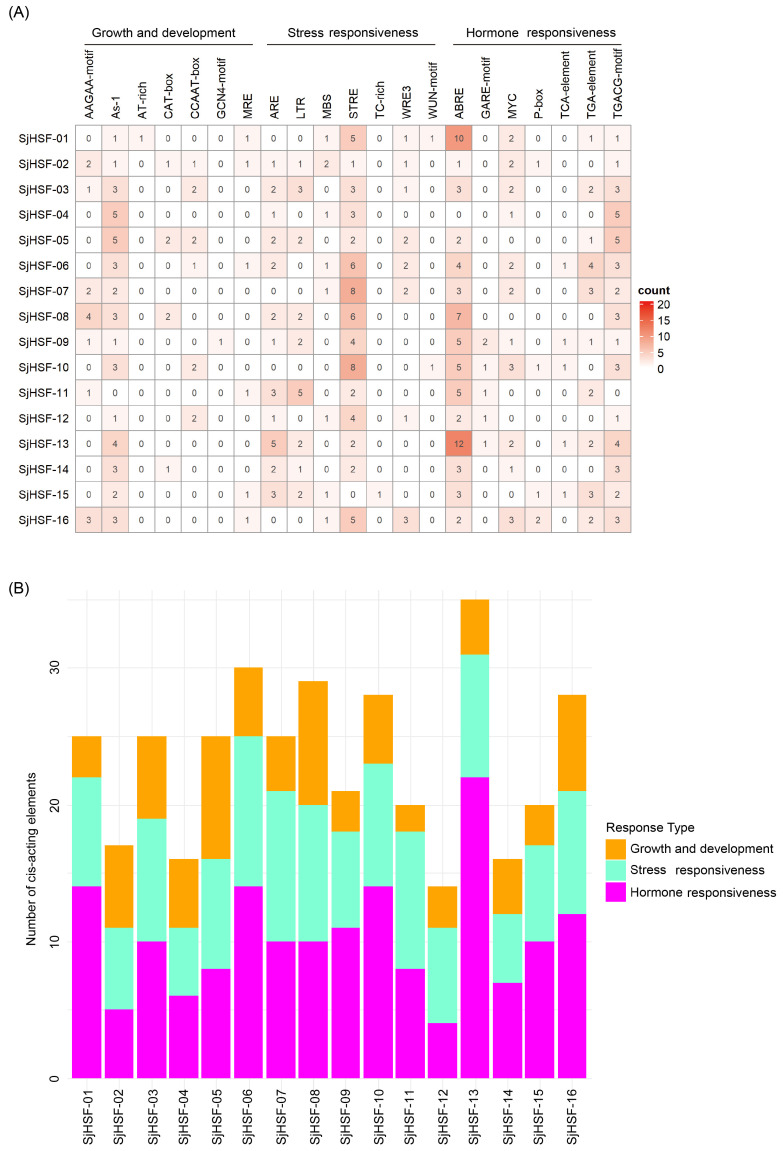
Summary of plant development, stress-inducible, and phytohormone cis-acting elements in the promoter regions of *SjHSF* genes. (**A**) The number of cis-acting elements in promoter regions of *SjHSF* genes. (**B**) The number of cis-acting elements in each category.

To explore the cis-acting regulatory elements in *SjHSFs*, the 2-kb upstream promoter region of each gene was analyzed using the PlantCARE database. Multiple cis-elements were identified and mainly classified into three groups: those associated with (1) plant growth and development, (2) stress response, and (3) hormone response. Among them, seven distinct stress responsive and seven hormone responsive elements were characterized (Figure 4A and Table S3). Each *SjHSF* contained at least one stress-responsive and hormone-responsive element, indicating their potential involvement in these signaling pathways. The most abundant elements were ABRE (67 instances) and STRE (61 instances). The primary hormone-responsive elements were ABRE, MYC, and TGACG-motif. In addition, development-related cis-elements, including GCN4-motif and AAGAA-motif (both linked to endosperm expression) and AT-box (linked to root and stem meristem expression), were detected in promoters of *SjHSFs* (Figure 4A). The abundance of hormone response elements exceeded that of stress response elements, suggesting *SjHSFs* may integrate hormone signaling to mediate responses to external environmental stresses (Figure 4B).

### 2.5. Tissue-Specific Expression of SjHSFs

**Figure 5:**
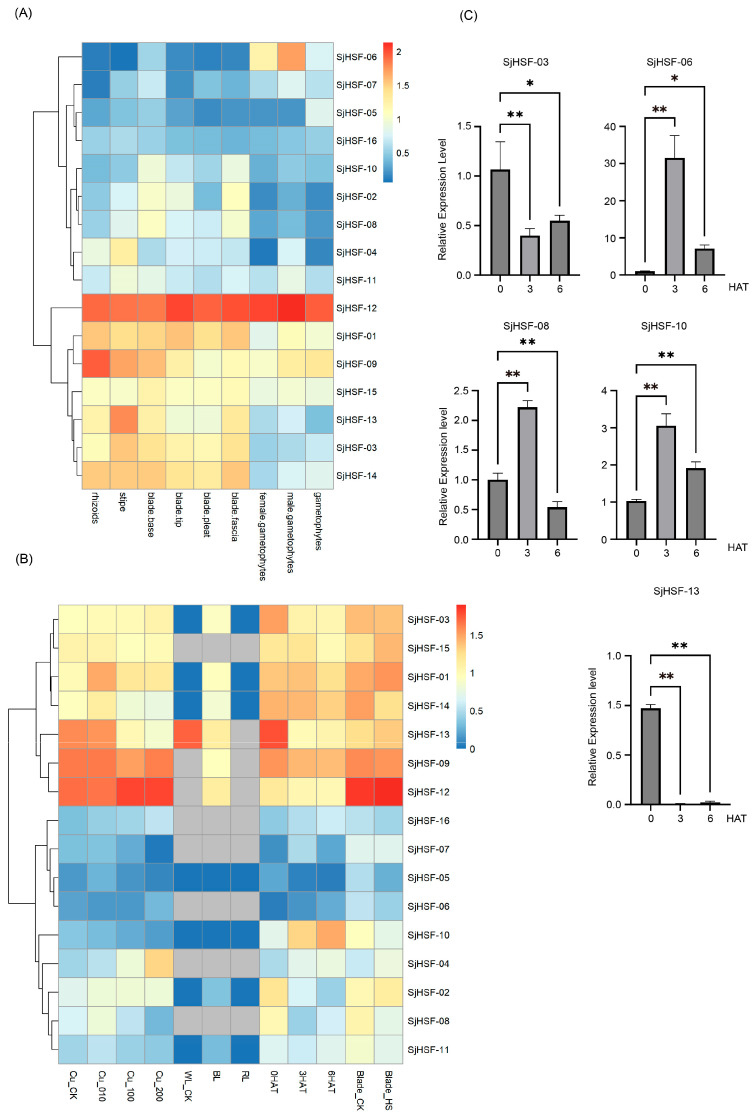
Heatmap of the expression patterns of *SjHSFs* during different stages and tissue sites (**A**) as well as under different stress treatments (**B**), and the expression profiles of five *SjHSFs* under heat stress by qRT-PCR validation (**C**). CK represents control; WL: white light; BL: blue light; RL: red light; HAT: heat after treatment; HS: hyposalinity. The red and blue colors represent higher and lower transcript levels compared to controls, respectively. Bars of qRT-PCR represent mean ± SD. Asterisks reveal the gene significantly upregulated or downregulated under heat stress by one-way ANOVA followed by Dunnett’s test (* *p* < 0.05; ** *p* < 0.01).

To explore the function of the *SjHSF* genes in *S. japonica* growth and development, tissue-specific expression levels were retrieved from public transcriptome data, covering rhizoids, stipes, blade bases, blade tips, blade pleats, blade fasciae, female gametophytes, and male gametophytes. All 16 *SjHSFs* exhibited variable expression across tissues (Figure 5A and Table S4). Specifically, seven genes (*SjHSF-01*, *-03*, *-09*, *-12*, *-13*, *-14*, and *-15*) were highly expressed in rhizoids, stipe, blade base, blade tip, blade pleat, and blade fascia tissues, implying roles in these specific tissues. *SjHSF-12* was highly expressed in all tissues, suggesting a broad role in *S. japonica* tissue development. *SjHSF-04* showed heightened expression exclusively in the stipe, indicating potential involvement in stipe-related processes. *SjHSF-06* was highly expressed in both female gametes and male gametophytes but low in other tissues, suggesting a specific function in gamete development.

### 2.6. Expression Patterns of SjHSFs Under Various Stresses

To explore the role of the *SjHSF* genes in abiotic stress responses, the expression profiles of these genes under copper ion stress, hyposalinity stress, different light treatments, and heat stress were analyzed using public RNA-seq data (Figure 5B and Table S5). The majority of *SjHSFs* exhibited no significant expression changes when exposed to different copper ion concentrations, with the exception of *SjHSF-01*, *-04*, and *-13*. Specifically, *SjHSF-01* displayed peak expression levels at 10 µg·L^−1^ Cu^2+^, while *SjHSF-04* was gradually up-regulated under different copper ion concentrations, and *SjHSF-13* was down-regulated (Figure 5B). As for different light treatments, *SjHSF-01*, *-03*, *-09*, *-12*, and *-14* exhibited increased expression levels under blue light, while *SjHSF-13* was down-regulated under blue light treatment. The majority of *SjHSFs* showed no significant change under hyposalinity stress; only *SjHSF-08* was down-regulated. Under heat stress, while approximately half of *SjHSFs* showed no significant expression changes, *SjHSF-04*, *-06*, *-10*, and *-16* were up-regulated, whereas *SjHSF-02*, *-03*, *-08*, and *-13* were down-regulated (Figure 5B and Table S6). Five representative genes (*SjHSF-03*, *-06*, *-08*, *-10*, and *-13*) were selected for qRT-PCR verification, and the results were largely consistent with RNA-seq data (Figure 5C and Figure S2, and Table S6).

### 2.7. Subcellular Localization of SjHSF-03 and Characterization of Conserved Motifs Recognized by SjHSF-03

**Figure 6:**
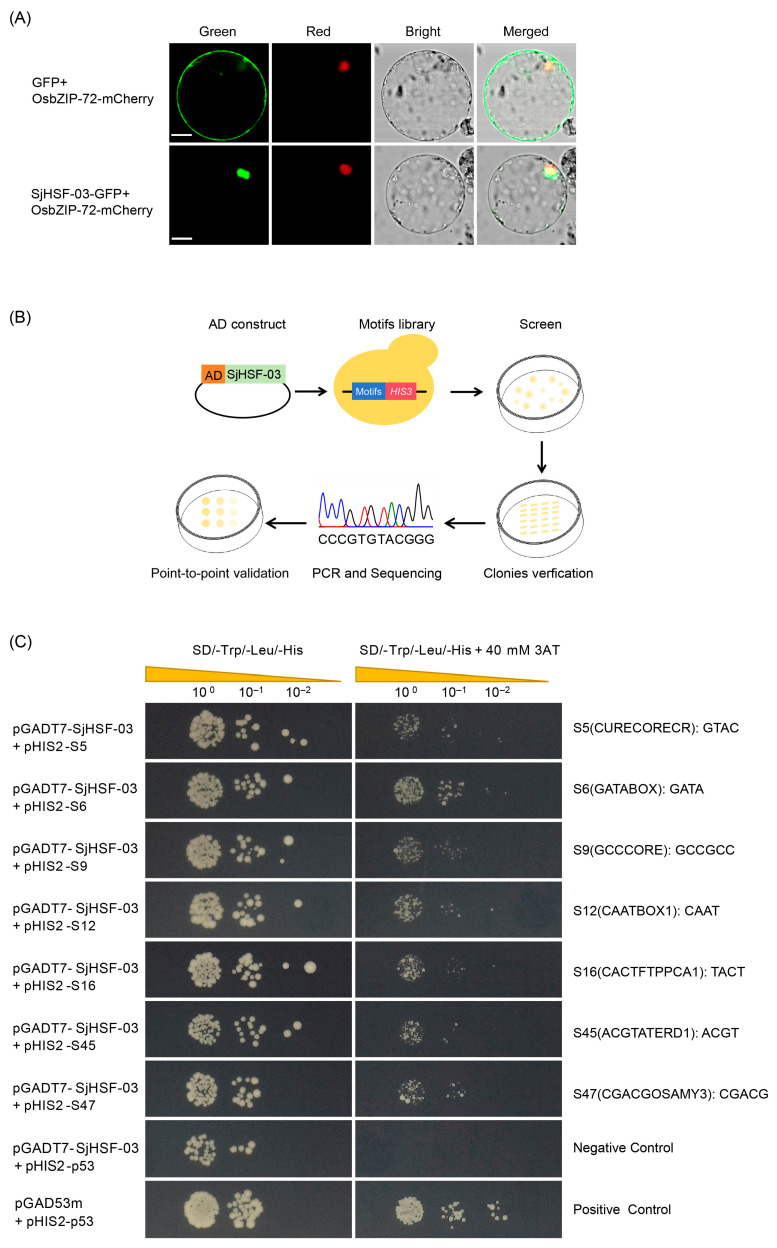
The molecular biology functions of SjHSF-03. (**A**) Subcellular localization of SjHSF-03 in rice protoplasts. OsbZIP72-mCherry was used as a nucleus marker. Scale bars = 50 μm. (**B**) TF-Centered Y1H workflow. (**C**) The binding of *SjHSF-03* to seven motifs. The transformants were screened on SD/-His/-Leu/-Trp medium and SD/-His/-Leu/-Trp medium with 40 mM 3-Amino-1, 2, 4-triazole (3-AT). p53HIS2/pGADT7-p53 and p53HIS2/pGADT7-SjHSF-03 were used as the positive control and negative control, respectively.

To further explore the functional properties of SjHSFs, SjHSF-03 was selected for the subcellular localization assay. Confocal imaging showed SjHSF-03 largely co-localized with the OsbZIP72 nuclear marker [25] (Figure 6A), confirming its nuclear localization.

A TF-Centered Y1H assay was performed to characterize the conserved motifs recognized by SjHSF-03 (Figure 6B). The results showed that SjHSF-03 could bind to seven conserved motifs, including CURECORECR (GTAC), GATABOX (GATA), GCCCORE (GCCGCC), CAATBOX1 (CAAT), CACTFTPPCA1 (YACT), ACGTATERD1 (ACGT), and CGACGOSAMY3 (CGACG) (Figure 6C; Table S7).

### 2.8. Distribution of SjHSF-03-Recognized Conserved Motifs in the Promoters of S. japonica Genes

**Figure 7:**
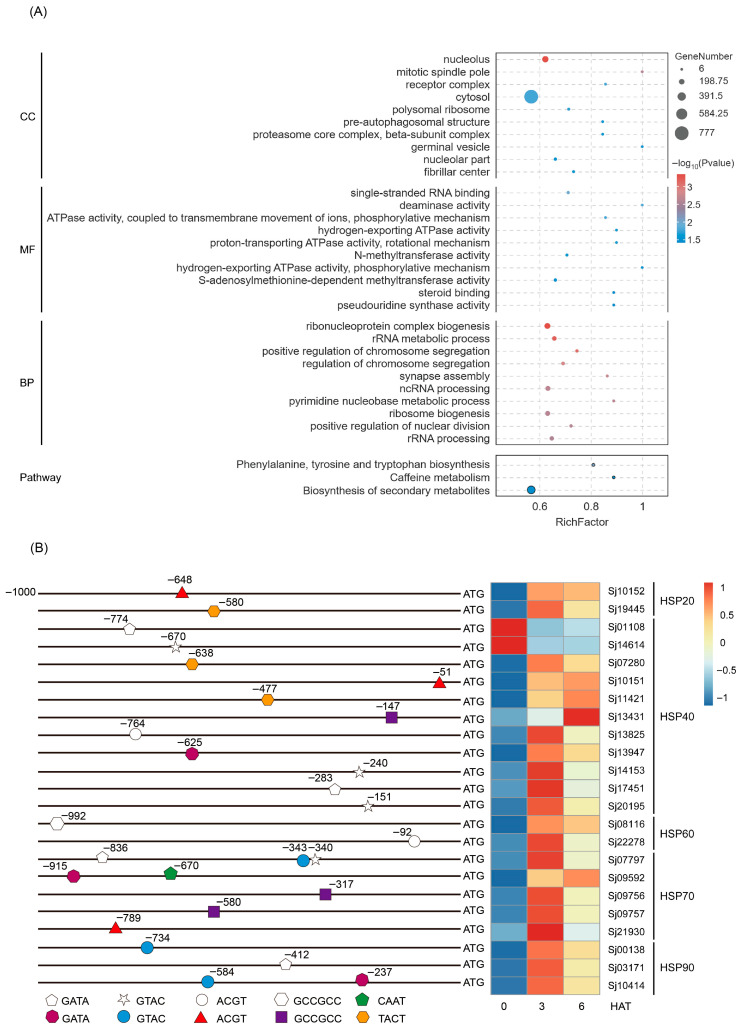
Go and KEGG enrichment analysis of the *SjHSF-03* potential target genes whose promoters contain one of seven motifs (**A**) and the position of motif in the *HSP* promoters and the heatmap showing the expression profiles of *HSPs* in response to heat stress (**B**). CC: Cellular component; MF: Molecular function; BP: Biological process.

Analysis of the distribution of the CURECORECR, GATABOX, GCCCORE, CAATBOX1, CACTFTPPCA1, ACGTATERD1, and CGACGOSAMY3 motifs in the promoters (−1 to −1000 bp) of *S. japonica* genes identified a total of 9970 genes (Table S8). These motif-containing genes were subjected to GO and KEGG enrichment analyses (Tables S9 and S10). In the GO “Cellular Component” category, these genes were primarily enriched in terms such as nucleolus, mitotic spindle pole, cytosol, etc. For the “Molecular Function” category, enrichment was observed in terms related to single-stranded RNA binding, deaminase activity, ATPase activity, methyltransferase activity, steroid binding, and pseudouridine synthase activity. In the “Biological Process” category, these genes clustered predominantly into terms associated with ribonucleoprotein complex biogenesis, rRNA metabolic process, chromosome segregation, synapse assembly, ribosome biogenesis, and ncRNA/rRNA processing (Figure 7A). Especially, two key stress-related GO terms involved in “positive regulation of stress-activated MAPK cascade” and “positive regulation of stress-activated protein kinase signaling cascade” (Table S9). Additionally, KEGG pathway enrichment analysis identified three significantly enriched pathways: phenylalanine, tyrosine and tryptophan biosynthesis; caffeine metabolism; and biosynthesis of secondary metabolites (Figure 7A). Overall, these results suggest that SjHSF-03 may target the promoter of genes involved in a wide range biological processes and signaling pathways.

Given the well-established role of heat shock proteins (HSPs) in stress responses, we further focused on identifying SjHSPs potentially regulated by SjHSF-03. A total of 23 *SjHSPs* were found to contain at least one of the SjHSF-03-recognized motifs (CURECORECR [GTAC], GATABOX [GATA], ACGTATERD1 [ACGT], GCCCORE [GCCGCC], CAATBOX1 [CAAT], or CACTFTPPCA1 [YACT]) in their promoters (Figure 7B). These *SjHSPs* belonged to five subfamilies: 2 *SjHSP20s*, 11 *SjHSP40s*, 2 *SjHSP60s*, 5 *SjHSP70s*, and 3 *SjHSP90s*. Notably, 21 out of these 23 *SjHSPs* (excluding 2 *SjHSP40s*) were up-regulated under heat stress (Figure 7B).

## 3. Discussion

HSFs are widely distributed in diverse organisms and play a crucial role in regulating abiotic stress responses and development [26]. Higher plants harbor a relatively higher number of HSFs. For example, *A. thaliana* possesses 21 HSFs [8]. A high abundance of HSFs has also been found in diatoms [27]. For example, 68 putative PtHSFs were identified from *P. tricornutum* [2]. By contrast, *HSF* gene numbers are more limited in other algal groups: the unicellular red alga *Cyanidioschyzon merolae* contains three *HSF* genes [18], while most red algae of the genus *Pyropia* (e.g., *Pyropia yezoensis*) have only one *HSF* gene [18]. Green algae typically encode one or two HSFs, and mosses (e.g., *Physcomitrium patens*) contain more than two [28]. In this study, we present the genome-wide identification and analysis of the *HSF* gene family in brown algae. About sixteen *SjHSF* genes were identified (Table 1), exceeding the number reported in the model moss *P. patens* (eight *HSFs*), suggesting *HSF* expansion occurred in *S. japonica*. For *S. japonica* inhabiting cold-temperate regions, low-temperature stress is a key abiotic factor shaping their distribution and survival. Thus, the *SjHSF* gene duplication is likely closely linked to their adaptive strategies in cold-temperate environments. However, their functions involved in cold tolerance need further study.

It is widely recognized that the DBD is an essential structural component of HSFs. The DBD exhibits a high degree of conservation, characterized by three α-helices and four β-turns [9]. The classification based on conserved motifs and domains suggests distinct regulatory mechanisms among SjHSFs, with most sharing the HSF DBD (Figure 1). All HSFAs feature activation domain motifs known as AHA motifs, which play a role in transcriptional activation [29]. All HSFBs, with the exception of HSFB5, have a tetrapeptide LFGV motif at the C-terminus [30]. NyHSF and CrHSF exhibit AHA and NES motifs, characteristic of the HSFA family [17,18]. In the *Arabidopsis* HSFA family, HSFA1 stands out as a key regulator that responds promptly to heat stress and activates other *HSF* genes such as *HSFA2*, *HSFA3*, *HSFA7*, *HSFB1*, and *HSFB2* [31]. The CrHSF1 protein constitutively forms trimers and is weakly expressed under non-stress conditions. However, its expression is rapidly induced upon heat shock [17]. Similarly, several HSFs in *S. japonica* also exhibit the characteristic features of plant HSFA. We identified eleven SjHSFs containing distinct AHA motifs that can be categorized as HSFA (Table S2), while no definitive LFGV tetrapeptide motif was found among the other HSFs. Further PCR amplification will be required to confirm the final gene structure of *SjHSFs*. Previous studies have indicated that all known algal HSFs fall into class A, possibly due to the stable thermal environments of marine and benthic habitats where algae predominantly inhabit, which may reduce the pressure for diversification into other HSF classes. PtHSF32 contains a unique AP2 region that is involved in responding to hormones and stress stimuli [2]. Interestingly, SjHSF-11 was identified as having a unique bZIP region in this study (Figure 1). The bZIP modulates hormone signaling pathways such as ABA and JA, and plays a regulatory role in plant drought and salt stress [32,33]. bZIP60 links the unfolded protein response and activates the expression of a type-A HSF, which subsequently upregulates the expression of *HSP* genes in response to heat stress in Maize [34]. Therefore, SjHSF-11 may also be involved in additional stress-response mechanisms. The HSF-HSP module plays a crucial role in hormone-mediated regulatory networks that regulate plant defensive response to various abiotic stresses [35].

Phylogenetic analysis revealed that SjHSFs with close evolutionary relationships share similar motif profiles (Figure 1). For example, motif 1 and motif 7 were present in most SjHSFs. In plants, gene regulation is frequently accomplished through the presence of introns [36,37]. Therefore, an analysis of intron-exon structures can provide valuable insights into gene function. It has been observed in red algae and diatom algae studies that these organisms typically possess fewer than three introns [2,18]. Our results show that all *SjHSFs*, except *SjHSF-07*, contain at least one intron (Figure 2). Notably, *SjHSF-11* contains up to 12 introns (Figure 2). The number of introns varies considerably among the 16 *SjHSFs*, which may contribute to their differential expression patterns across tissues. Phylogenetic analysis revealed that brown algal HSFs form an independent clade, supporting lineage-specific evolution (Figure 3). A notable morphological distinction lies in the chloroplast membrane structure: chloroplasts of red and green algae, which are closely related to land plants, are surrounded by two membranes [38], whereas those of brown algae, which diverged earlier in evolution, are enclosed by four membranes [39].

HSFs enhance plant resilience to abiotic stress through modulation of gene expression [40]. Research indicates that temperature serves as the primary environmental factor affecting algal developmental processes, including the induction of gametes [22]. Transcriptomic analysis of *S. japonica* under heat stress conditions revealed eight differentially expressed *SjHSF* genes (*SjHSF-02*, *-03*, *-04*, *-06*, *-08*, *-10*, *-13*, and *-16*) (Table S6). Notably, our previous study employed more stringent selection criteria, detected only a single *HSF* gene (*SjHSF-10*) as differentially expressed with NR annotation [41]. This suggests that the current experimental approach may have enhanced sensitivity in identifying heat-responsive transcriptional regulators. Among them, three DEGs (*SjHSF-04*, *-08*, and *-13*) also exhibited differential regulation under copper ions (Cu^2+^) stress. In addition, the expression level of *SjHSF-04* was up-regulated upon thermal and copper ion stress conditions. Heavy metal stress response pathways are activated upon detection of Cu^2+^ ions, involving signaling molecules such as calcium-dependent protein kinases (CDPKs) and mitogen-activated protein kinases (MAPKs), which subsequently modulate various metal-responsive TF families in kelp, including NAC and HSFs [42,43]. Among the eight representative *SjHSFs*, only *SjHSF-13* was down-regulated under blue light treatment, whereas the expression levels of the remaining *SjHSFs* were nearly undetectable under light stress conditions. In summary, the differential expression of *SjHSFs* under abiotic stresses indicates their potential involvement in the stress response mechanisms of *S. japonica*.

In the present study, SjHSF-03 was selected to investigate the functional characteristics. Subcellular localization analysis via transient expression in rice protoplasts revealed that SjHSF-03 is predominantly localized to the nucleus (Figure 6A), consistent with the canonical features of transcription factors. Results from the TF-Centered Y1H assay demonstrated that SjHSF-03 can bind to seven conserved motifs: CURECORECR (GTAC), GATABOX (GATA), GCCCORE (GCCGCC), CAATBOX1 (CAAT), CACTFTPPCA1 (YACT), ACGTATERD1 (ACGT), and CGACGOSAMY3 (CGACG) (Figure 6C; Table S7). These motifs are known to mediate diverse biological processes, including stress responses, hormone signaling, and growth and development—suggesting that SjHSF-03 may regulate multiple physiological pathways. Consistent with this, we identified a total of 9970 *S. japonica* genes whose promoters harbor one or more of the seven motifs bound by SjHSF-03. Functional annotation analysis indicated that these potential target genes participate in a broad spectrum of biological processes and signaling cascades (Figure 7A). Notably, the promoters of 23 *SjHSPs* contain the conserved motifs recognized by SjHSF-03. While SjHSF-03 was down-regulated under heat stress, 21 of these 23 *SjHSPs* were up-regulated (Figure 7B). These observations suggest that SjHSF-03 may act as a negative transcription factor in the heat stress response. Hormone-mediated regulatory networks involved in heat tolerance reveal the importance of the HSF-HSP module and its upstream regulatory mechanisms [35]. Nonetheless, additional experiments (e.g., overexpression, knockdown, or genome editing) are required to validate and extend these findings in future studies.

## 4. Materials and Methods

### 4.1. Identification and Analysis of HSFs in S. japonica

The genomic, cDNA, and protein sequences of *S. japonica* were downloaded from ORCAE (https://bioinformatics.psb.ugent.be/orcae/overview/Sacja; accessed on 8 September 2025) and GCA_048937375.1 [23]. To identify the members of the *SjHSF* gene family, the hidden Markov model file of HSF domain (PF00447) was downloaded from the Pfam database (http://pfam.xfam.org/; accessed on 30 October 2025) and used as a query in a HMMER (e-value < 1 × 10^−5^) search against the *S. japonica* protein database (https://www.ebi.ac.uk/Tools/hmmer/search/phmmer; accessed on 16 January 2026). The sequences of SjHSF-like proteins (thereafter referred to as SjHSFs) were further analyzed using the SMART (https://www.ebi.ac.uk/Tools/hmmer/; accessed on 30 October 2025) and InterPro databases (https://www.ebi.ac.uk/interpro/search/sequence/; accessed on 30 October 2025) to verify the presence of the HSF domain.

### 4.2. Multiple Sequence Alignment and Phylogenetic Analysis

We constructed two phylogenetic trees, one with SjHSF protein sequences and the other including HSF protein sequences from multiple additional species. The HSF protein sequences of *Physcomitrium patens* and *Ectocarpus siliculosus* were obtained from ORCAE (https://bioinformatics.psb.ugent.be/orcae/; accessed on 8 September 2025), whereas sequences of *A. thaliana* and *C. reinhardtii* were retrieved from the Phytozome database (https://phytozome-next.jgi.doe.gov; accessed on 8 September 2025). Additionally, the sequences of *Cyanidioschyzon merolae*, *Pyropia yezoensis*, and *Pyropia umbilicalis* were acquired from the NCBI database (https://www.ncbi.nlm.nih.gov/; accessed on 8 September 2025). *Klebsormidium flaccidum* and *Volvox carteri* were downloaded from TDFB (https://planttfdb.gao-lab.org/; accessed on 8 September 2025). Multiple sequence alignment of the full predicted HSF protein sequences was performed using Muscle in Molecular Evolutionary Genetics Analysis (MEGA) 11.0 software with default parameters. The alignments were refined with MEGA11.0 usingClustalw method. Subsequently, phylogenetic trees were reconstructed using the Maximum Likelihood (ML) method in MEGA11.0. Branch support was evaluated with 1000 bootstrap replicates, and gaps were handled using the pairwise deletion option.

### 4.3. Sequence Analysis, Structural Characterization, and Chromosome Localization

The isoelectric point (pI), number of amino acids, and molecular weight (MW) of proteins were calculated using ExPasy (http://web.expasy.org/; accessed on 30 October 2025). The subcellular localization of SjHSFs was predicted using Euk-mPLoc 2.0 server (http://www.csbio.sjtu.edu.cn/bioinf/euk-multi-2/; accessed on 30 October 2025) and WoLF PSORT (https://www.genscript.com/wolf-psort.html/; accessed on 30 October 2025). Motifs of SjHSFs were analyzed by the MEME tool (http://meme-suite.org/tools/meme; accessed on 30 October 2025) with default parameters. Analysis of the *SjHSF* gene structure was carried out by comparison of the coding sequences (CDS) and genomic DNA sequences using GSDS2.0 software (https://gsds.gao-lab.org; access on 30 October 2025). Protein domains of SjHSFs were annotated using the InterPro database and visually represented using Jalview2.10.4b1 software. The TBtools-IIv2.390 tool was used to determine the chromosomal locations of the *SjHSF* genes to identify their precise chromosomal position in the genome [23].

### 4.4. Promoter Analysis of SjHSFs

The 2.0 kb upstream promoter region relative to the translation initiation codon ATG of each *SjHSF* gene was subjected to the PlantCARE database (http://bioinformatics.psb.ugent.be/webtools/plantcare/html/; accessed on 30 October 2025) to identify cis-elements. The expression heatmap of cis-acting elements was generated using the pheatmap package in R. Additionally, a bar chart illustrating the distribution of cis-acting elements was generated using R4.4.3 software.

### 4.5. Transcriptome Analysis of the SjHSF Gene Across Different Tissues and Under Stress Conditions

Transcriptome data of *SjHSF* genes in different tissues and under hyposalinity stress were retrieved from the National Genomics Data Center (accession: PRJCA000815) [44]. To investigate the expression patterns of *SjHSF* genes under abiotic stress conditions, the related RNA-seq datasets were obtained from the NCBI database, including those for heat stress (PRJNA949272), light stress (SRA049951), and copper stress (PRJNA387211) [41,45,46]. The treatment conditions for relevant stresses were provided as followed: (1) the young sporophytes subjected to 25 °C were harvested after different times (0, 3, and 6 h); (2) the young sporophytes were cultured under a white light intensity of 35 ± 5 μmol m^−2^ s^−1^ with a 10/14 h light/dark cycle. Following a 24-h period of darkness, the samples were then cultured under continuous red light (RL), blue light (BL), and white light (WL) at the same intensity of 35 ± 5 μmol m^−2^ s^−1^; (3) the young sporophytes were cultured in seawater with 10, 100, and 200 μg L^−1^ Cu^2+^, respectively. The data of RNA-seq was analyzed using hisat2 [47] with transcripts per million (TPM) used for normalization. Different expression genes were identified using the thresholds |log2FC| > 1, *p* value < 0.05. Expression heatmaps were constructed using R software, with the log_10_ (TPM + 1) values of gene expression in different treatments.

### 4.6. RNA Extraction and Quantitative RT-PCR

Total RNA was isolated from *S. japonica* samples subjected to 25 °C thermal stress for 0, 3, and 6 h using the Trizol method. Next, first-strand cDNA was synthesized using HiScript^®^ III RT SuperMix for qPCR (+gDNA wiper) Kit (Vazyme Biotech, Nanjing, China). The qRT-PCR analysis was performed using SYBR Green Master Mix (Vazyme Biotech, Nanjing, China). The *β-actin* gene was used as an internal control. Relative gene expression values were calculated via the 2^−△△Ct^ method [48]. The primers used for qRT-PCR are listed in Table S11. All data are obtained from statistical analysis of three repeats.

### 4.7. Subcellular Localization of SjHSF-03

To verify the prediction of subcellular localization, a protoplast system was used for transient expression analysis of the pRTVcGFP vector [49]. The full-length CDS of a representative *SjHSF-03* gene was constructed into the PRTVcGFP vector to fuse in-frame with *GFP*. *Oryza sativa* L. ssp. *japonica* was used in this study. The 14-day-old etiolated seedlings for prepared rice protoplasts were cultured under a photoperiod of 24 h dark in 28 °C. Rice protoplasts were transformed via the polyethylene glycol (PEG)-mediated method [50].

### 4.8. TF-Centered Y1H Screening

A random short DNA sequence insertion library was generated following the protocol [51] to serve as the prey library at ProNet Biotech Co., Ltd. (Nanjing, China). The coding sequence(CDS) of *SjHSF-03* was inserted into pGADT7 vector as the bait. Y1H screening was performed to identify cis-acting elements bound by SjHSF-03. According to high-stringency selection, the positive clones were sequenced to analyze the inserts in the pHIS2 plasmids. The insertion sequences, along with their left and right insertion flanking sequences (“GGG” and “CCC”), were analyzed to determine whether they are known cis-acting elements using the PlantCARE (http://bioinformatics.psb.ugent.be/webtools/plantcare/html/; accessed on 21 November 2025), a specialized resource for plant promoter analysis and cis-acting regulatory element identification.

To investigate whether SjHSF-03 binds to the predicted DNA motifs, the CDS of SjHSF-03 was inserted into the pGADT7 vector to generate the bait construct, while sequences containing known cis-acting elements identified in the screening served as preys. The preys were individually co-transformed with the bait vector into the strain yeast Y187 (MATα). The interactions between conserved motifs and SjHSF-03 were point-to-point validated on SD/-His/-Leu/-Trp or SD/-His/-Leu/-Trp medium supplied with 3-AT (3-Amino-1, 2, 4-triazole). The interactions between pHIS2-p53, which consists of three tandem copies of the cis-acting DNA consensus sequence inserted into the multiple cloning site (MCS) of pHIS2 and is specifically recognized by p53, were employed as negative controls for the tested SjHSF-03.

### 4.9. Gene Ontology (GO) and Kyoto Encyclopedia of Genes and Genomes (KEGG) Analysis

Gene Ontology (GO) and Kyoto Encyclopedia of Genes and Genomes (KEGG) pathway enrichment analyses were conducted using OmicShare Tools [52].

## 5. Conclusions

This study characterized the *HSF* gene family in *S. japonica*, identifying 16 *SjHSF* genes. Phylogenetic analysis revealed that brown algae HSFs exhibit a distinct evolutionary relationship compared to those in green algae, red algae, and land plants. The DBD of SjHSFs was highly conserved, consistent with its role in HSF-mediated transcriptional regulation. The expression of *SjHSFs* varies in different tissues and upon abiotic treatments. SjHSF-03 is localized to the nucleus and can bind to seven conserved motifs. A total of 9970 *S. japonica* genes harbor the SjHSF-03-recognized motifs in their promoter, including 23 *SjHSP* genes. Under heat stress, 21 of these 23 *SjHSPs* were up-regulated, while *SjHSF-03* expression was down-regulated. Collectively, these results provide critical insights into the functional characteristics of SjHSF-03 and lay a foundation for further elucidating the regulatory networks of HSFs in *S. japonica*.

## Figures and Tables

**Table 1:** Identification and characterization of sixteen *HSF* genes in *S. japonica*.

Gene Name	Guo et al., 2025 [23]		Ye et al., 2015 [24]		CDS Length	Size (aa)	MW (kDa)	pI	Cellular Location	Instability Index	Family
	Chromosome	Location Coordinates (5′–3′)	Chromosome	Location Coordinates (5′–3′)							
*SjHSF-01*	chr3	16,226,480 –16,234,914	chr0	111,944,806–111,951,284	1791	596	61.64	4.98	Nucleus	53.89	HSFA
*SjHSF-02*	chr16	1,276,777–1,285,814	chr23	12,138,926–12,144,645	2277	758	79.06	10.06	Nucleus	67.47	-
*SjHSF-03*	chr24	9,363,405–9,375,594	chr0	110,362,099–110,374,190	2043	680	71.34	6.41	Nucleus	62.16	-
*SjHSF-04*	chr24	9,851,910–9,862,767	chr0	175,525,224–175,533,393	2559	852	84.52	6.08	Nucleus	54.74	HSFA
*SjHSF-05*	chr25	6,852,895–6,855,268	chr5	11,698,548–11,734,512	1191	396	43.08	8.87	Nucleus	54.97	HSFA
*SjHSF-06*	chr25	5,120,112–5,125,129	chr5	9,221,350–9,246,951	2553	850	94.01	9.42	Nucleus	55.41	-
*SjHSF-07*	chr32	6,923,666–6,944,073	chr0	60,759,876–60,760,284	411	136	14.75	6.85	Nucleus	79.62	-
*SjHSF-08*	chr10	884,604–900,978	chr22	8,626,351–8,646,018	3978	1325	131.25	9.34	Nucleus	55.31	HSFA
*SjHSF-09*	chr16	1,735,269–1,750,050	chr23	11,418,163–11,430,094	2073	690	72.05	7.11	Nucleus	67.45	HSFA
*SjHSF-10*	chr11	10,621,736–10,633,647	chr9	5,349,005–5,353,569	2343	780	76.13	4.99	Nucleus	49.46	HSFA
*SjHSF-11*	chr14	14,684,095–14,708,999	chr2	9,896,580–9,923,402	2745	914	88.81	5.03	Nucleus	38.26	-
*SjHSF-12*	chr16	3,740,072–3,748,913	chr23	9,340,684–9,347,948	1896	631	62.30	10.82	Nucleus	52.82	HSFA
*SjHSF-13*	chr6	16,794,337–16,809,476	chr11	11,801,494–11,819,000	2874	957	98.27	8.56	Nucleus	63.2	HSFA
*SjHSF-14*	chr28	1,786,382–1,807,750	chr19	1,945,525–1,965,480	2388	795	82.53	7.18	Nucleus	59.16	HSFA
*SjHSF-15*	chr20	7,379,369–7,405,020	chr0	56,040,433–56,058,278	3252	1083	112.28	5.94	Nucleus	51.5	HSFA
*SjHSF-16*	chr6	1,081,980–1,092,458	chr0	42,671,959–42,676,596	1839	612	62.19	9.35	Nucleus	49.2	HSFA

Note: “-“ denotes unconfirmed.

## Data Availability

The RNA-seq data generated in this study are available in the NCBI using accession numbers PRJNA949272, PRJNA387211, SRA049951, and National Genomics Data Center Data accession numbers PRJCA000815 will be made available on request.

## Supplementary Materials

The following supporting information can be downloaded at: https://www.mdpi.com/article/10.3390/plants15030429/s1, Table S1. Sequence of sixteen *HSFs* identified in *S. japonica*; Table S2. Conserved motifs in the AHA of SjHSFs; Table S3. Cis-elements of *SjHSFs*; Table S4. The expression levels of *SjHSF* genes during different tissues and development stages in *S. japonica*; Table S5. The expression levels of *SjHSF* genes response to various stress treatments in *S. japonica*; Table S6. Eight DEGs from sixteen *SjHSFs* identified under heat stress in *S. japonica*; Table S7. Analysis of the insertion sequences that bind to SjHSF-03; Table S8. Analysis of the distribution of the CURECORECR, GATABOX, GCCCORE, CAATBOX1, CACTFTPPCA1, ACGTATERD1, and CGACGOSAMY3 motifs in the promoters (−1 to −1000 bp) of *S. japonica* genes. Table S9. GO enrichment analysis of potential target genes; Table S10. Significant KEGG pathways of potential target genes; Table S11. Primers used in this study. Figure S1. HR-A/B domain analysis of the SjHSFs. Figure S2. Comparison between RNA-seq and qRT-PCR.

## Abbreviations

The following abbreviations are used in this manuscript:

BP	Biological process
CC	Cellular components
CDS	Coding sequences
CTAD	C-terminal transcriptional activation domain
DBD	DNA-binding domain
GO	Gene Ontology
HS	Heat shock
HSF	Heat shock transcription factor
HSP	Heat shock protein
KEGG	Kyoto Encyclopedia of Genes and Genomes
MEGA	Muscle in Molecular Evolutionary Genetics Analysis
MF	Molecular functions
MW	Molecular weight
NES	Nuclear output signal
NJ	Neighbor-Joining
NLS	Nuclear localization signal
OD	Oligomerization domain
pI	Isoelectric point
TF-Centered Y1H	Transcription Factor-Centered Yeast one Hybrid
TPM	Transcripts per million
