# Supplementary figures and images for: Genome-Wide Analysis of Heat Shock Transcription Factors (HSFs) in Kelp (Saccharina japonica) and Analysis of Their Expression in Response to Abiotic Stresses
Source: Plants (Basel). 2026 Jan 30;15(3):429. doi: 10.3390/plants15030429 (PMC12899012; doi:10.3390/plants15030429)

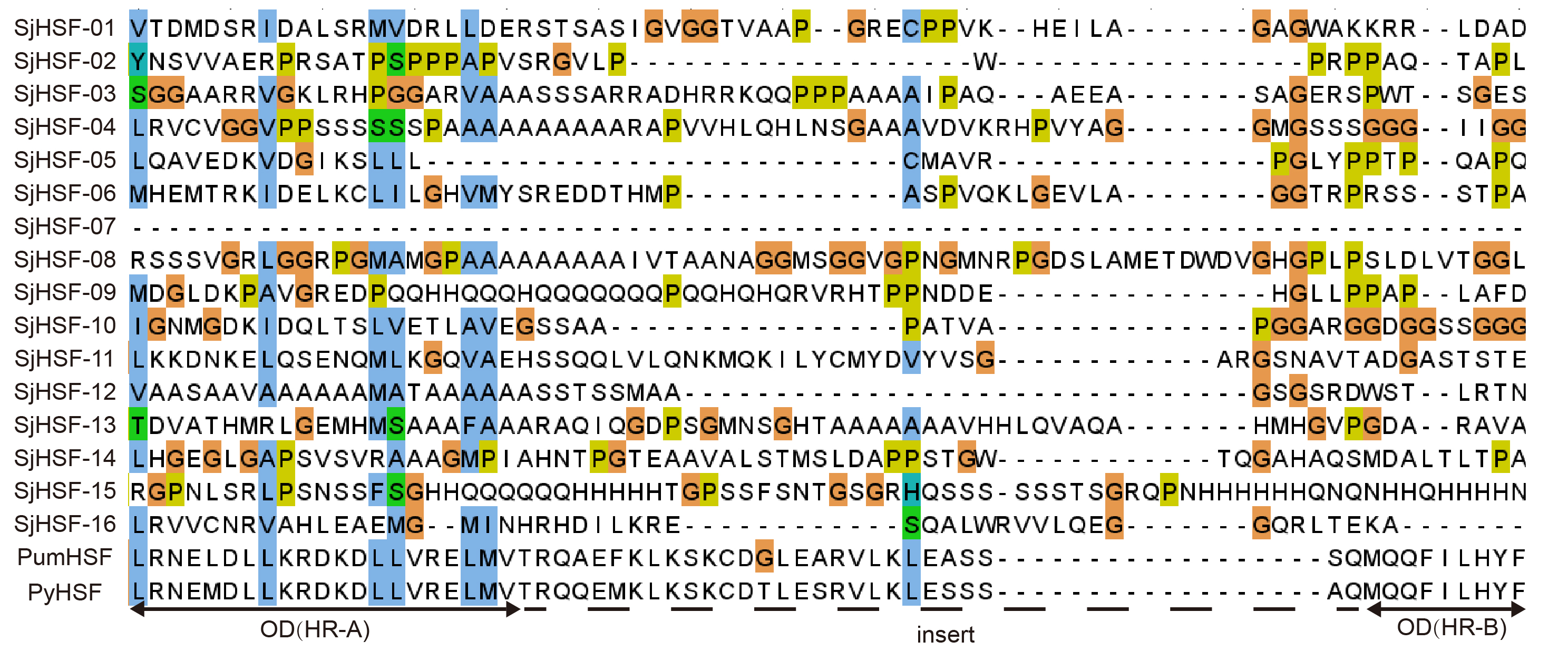

Supplement: Supplementary file 1 [file plants-15-00429-s001.zip › Figure S1- domain.jpg]

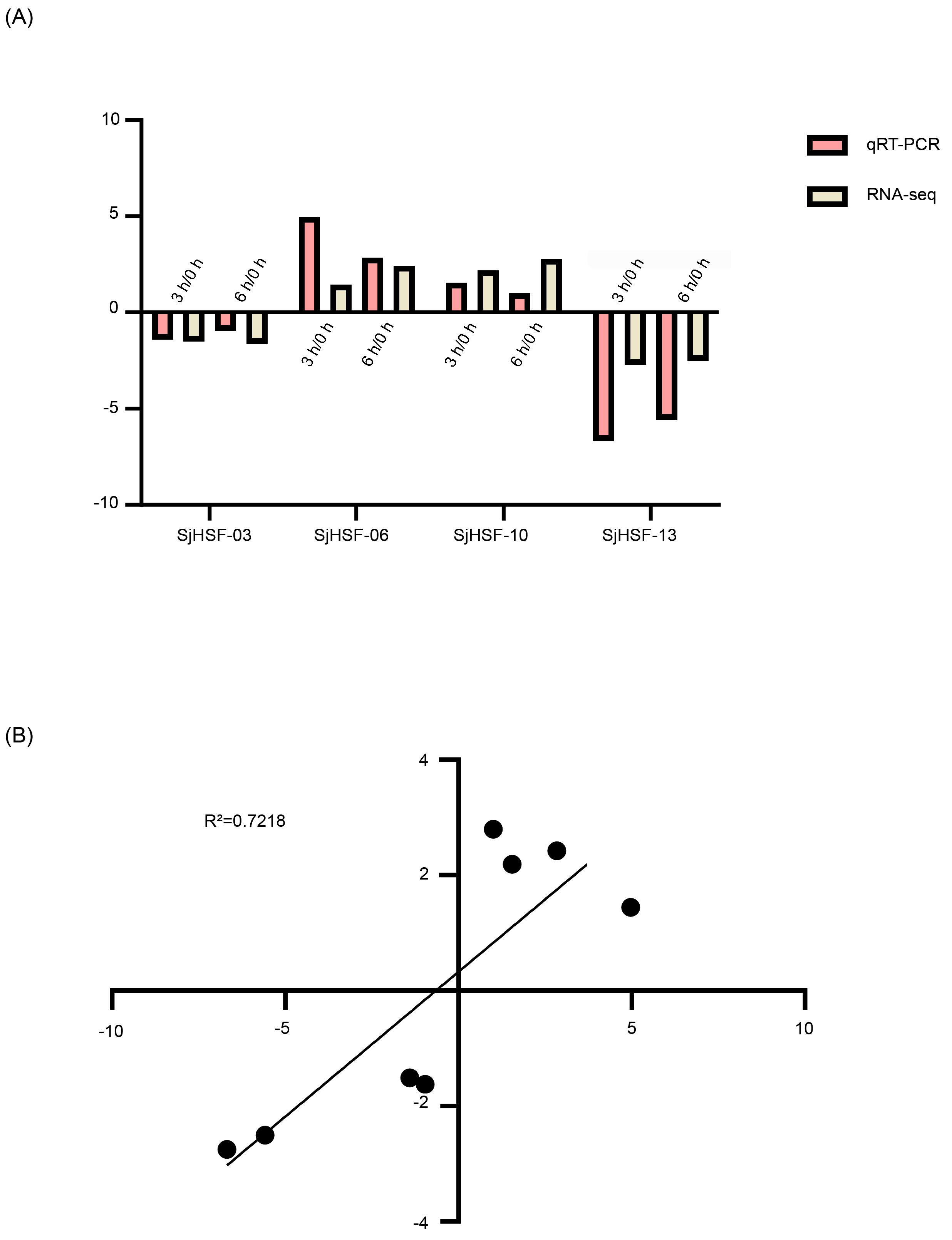

Supplement: Supplementary file 1 [file plants-15-00429-s001.zip › Figure S2.jpg]
